# Supplementary material for: Which Suture to Choose in Hepato-Pancreatic-Biliary Surgery? Assessment of the Influence of Pancreatic Juice and Bile on the Resistance of Suturing Materials—In Vitro Research
Source: Biomedicines. 2022 May 2;10(5):1053. doi: 10.3390/biomedicines10051053 (PMC9139064; doi:10.3390/biomedicines10051053)
Supplement: Supplementary file 1 [file biomedicines-10-01053-s001.zip › biomedicines-1676877-supplementary.pdf]

biomedicines

Supplementary Material

Which Suture to Choose in Hepato-Pancreatic-Biliary Surgery? Assessment of the Influence of Pancreatic Juice and Bile on the Resistance of Suturing Materials—In Vitro Research

Marcin Gierek [1,*], Katarzyna Merkel [2,*], Gabriela Ochała-Gierek [3], Paweł Niemiec [4], Karol Szyluk [5,6] and Katarzyna Kuśnierz [7]

[1] Center for Burns Treatment im. Dr Sakiel, ul. Jana Pawła II 2, 41-100 Siemianowice Śląskie, Poland; marcin.gierek@clo.com.pl
[2] Institute of Materials Engineering, Faculty of Science and Technology, University of Silesia, ul. 75. Pułku Piechoty, 41-500 Chorzów, Poland; Katarzyna.merkel@us.edu.pl
[3] Dermatology Department, City Hospital in Sosnowiec, ul. Zegadłowicza 3, 41-200 Sosnowiec, Poland; g.ochala@wp.pl
[4] Department of Biochemistry and Medical Genetics, Faculty of Health Sciences in Katowice, Medical University of Silesia in Katowice, 40-752 Katowice, Poland; pniemiec@sum.edu.pl
[5] Department of Physiotherapy, Faculty of Health Sciences in Katowice, Medical University of Silesia in Katowice, 40-752 Katowice, Poland; karol.szyluk@sum.edu.pl
[6] Department of Orthopaedic and Trauma Surgery, District Hospital of Orthopaedics and Trauma Surgery, 41-940 Piekary Śląskie, Poland
[7] Department of Gastrointestinal Surgery, Medical University of Silesia in Katowice, ul. Medyków 14, 40-752 Katowice, Poland; kasiachir@wp.pl
* Correspondence: marcin.gierek@clo.com.pl (M.G.); Katarzyna.merkel@us.edu.pl (K.M.); Tel.: +486-6070-7704 (M.G.); +486-9834-2856 (K.M.).

Citation: Gierek, M.; Merkel, K.; Ochała-Gierek, G.; Niemiec, P.; Szyluk, K.; Kuśnierz, K. Which Suture to Choose in Hepato-Pancreatic-Biliary Surgery? Assessment of the Influence of Pancreatic Juice and Bile on the Resistance of Suturing Materials—In Vitro Research. *Biomedicines* **2022**, *10*, 1053. https://doi.org/10.3390/biomedicines10051053

Academic Editor: Mike Barbeck

Received: 27 March 2022
Accepted: 30 April 2022
Published: 2 May 2022

Publisher's Note: MDPI stays neutral with regard to jurisdictional claims in published maps and institutional affiliations.

Copyright: © 2022 by the authors. Licensee MDPI, Basel, Switzerland. This article is an open access article distributed under the terms and conditions of the Creative Commons Attribution (CC BY) license (https://creativecommons.org/licenses/by/4.0/).

1. BASE STATE ASSESSMENT (BRAND NEW SURGICAL SUTURES)

Table S1. Amylase and lipase levels in pancreatic juice before freezing and after thawing along with pH measurements of pancreatic juice and bile.

Nr	Amylase (before freezing) U/l	Amylase (after thawing) U/l	Lipase (before freezing) U/l	Lipase (after thawing) U/l	pH pancreatic juice	pH bile
1	157 400	138 920<br>122 700	789 200	722 050<br>693 050	+/- 7,7	+/- 6,8
2	66 440	40 900	392 400	196 200		
3	84 100	78 560	702 600	577 000		
4	21 260	20 260	84 590	84 060		

There was a slight decrease in amylase and lipase levels after thawing (mean decrease for amylase = 16.48% and for lipase = 17.64%).

Table S2. Assessment of the tensile strength of the reference surgical sutures used in the test (brand new threads).

REFERENCE STATE											
**Tensile Srength R_m (MPa)**	**arithmetic average**	**Median**	**Min.**	**Max.**	**Lower Quartile**	**Upper Quartile**	**Standard deviation**	**Standard Error**	**Skewness**	**Kurtosis**	**Test Shapiro-Wilka**
MONOCRYL	236.5	238.1	211.6	273.9	217.4	239.8	21.92	8.95	0.8556	1.2525	p = 0,3933
MONOCRYL Plus	268.7	268.9	222.3	305.0	251.0	296.0	31.14	12.71	-0.3661	-0.9902	p = 0,7751
PDS	117.2	112.7	109.6	132.5	110.5	125.0	9.38	3.83	1.1627	-0.3114	p = 0,0727
PDS Plus	127.8	127.4	116.4	138.7	122.6	134.4	8.26	3.37	-0.0489	-1.0993	p = 0,9342
VICRYL	293.0	305.8	238.9	320.2	281.0	306.3	29.37	11.99	-1.5789	2.4181	p = 0,0864
VICRYL Plus	282.8	278.1	262.6	315.0	263.8	299.3	22.11	9.03	0.4988	-1.7340	p = 0,1953

- Levene's test showed homogeneity of variance: p = 0.0511
- ANOVA test showed a statistically significant difference in the Rm level between the study groups (p <0.0001)

Table S3. ANOVA statistical test results for the reference tensile strength (brand new threads): SS - sum of squares, MS - sum of mean squares, F - F index (F distribution)

ANOVA TEST	SS	Degrees of freedom	MS	F	p
**Intercept**	1758079	1	1758079	3565.931	< 0.0001
**Material**	185850	5	37170	75.392	**< 0.0001**
**Error**	14791	30	493		

Table S4. Results of Tukey's post-hoc tensile strength test for reference suture state.

Material	MONOCRYL	MONOCRYL Plus	PDS	PDS Plus	VICRYL	VICRYL Plus
**MONOCRYL**	p =	0.1523	**0.0001**	**0.0001**	**0.0017**	**0.0127**
**MONOCRYL Plus**	0.1523	p =	**0.0001**	**0.0001**	0.4248	0.8768
**PDS**	**0.0001**	**0.0001**	p =	0.9590	**0.0001**	**0.0001**
**PDS Plus**	**0.0001**	**0.0001**	0.9590	p =	**0.0001**	**0.0001**
**VICRYL**	**0.0017**	0.4248	**0.0001**	**0.0001**	p =	0.9666
**VICRYL Plus**	**0.0127**	0.8768	**0.0001**	**0.0001**	0.9666	p =

- Tukey's post-hoc test showed a statistically significant difference in the Rm level between:
MONOCRYL vs. PDS;
MONOCRYL vs. PDS Plus;
MONOCRYL vs. VICRYL;
MONOCRYL vs. VICRYL Plus;
MONOCRYL Plus vs. PDS;
MONOCRYL Plus vs. PDS Plus;
PDS vs. VICRYL;
PDS vs. VICRYL Plus;
PDS Plus vs. VICRYL;
PDS Plus vs. VICRYL Plus.

2. EVALUATION OF THE INFLUENCE OF THE EXPOSURE TIME ON THE TENSILE STRENGTH (Rm) - STERILE ENVIRONMENT

Table S5. Results of tensile strength tests of Monocryl sutures in saline (sterile environment).

MONOCRYL	SALINE - STERILE ENVIRONMENT										
**Tensile Srength R_m (MPa)**	**arithmetic average**	**Median**	**Min.**	**Max.**	**Lower Quartile**	**Upper Quartile**	**Standard deviation**	**Standard Error**	**Skewness**	**Kurtosis**	**Test Shapiro-Wilka**
**Reference**	236.5	238.1	211.6	273.9	217.4	239.8	21.92	8.95	0.8556	1.2525	p = 0,3933
**7 days**	132.7	136.2	113.5	149.4	116.8	143.9	14.58	5.95	-0.4545	-1.6955	p = 0,4524
**14 days**	93.0	94.0	78.0	112.5	81.3	98.1	12.59	5.14	0.4015	-0.1993	p = 0,7746
**21 days**	35.3	35.7	30.4	41.2	32.0	36.6	3.79	1.55	0.3312	0.1625	p = 0,8155
**28 days**	6.9	7.1	5.7	7.7	6.5	7.4	0.69	0.28	-0.9257	0.5636	p = 0,6697

- The ANOVA test showed a statistically significant difference in the Rm level between all exposure times (**p <0.0001**) (i.e. statistically significant dynamics of Rm changes)
- Tukey's post-hoc test showed a statistically significant difference in the Rm level.
There is a statistically significant decrease in the Rm level with the lapse of the exposure time

Table S6. Results of tensile strength tests of Monocryl sutures in the pancreatic juice (sterile environment).

MONOCRYL	PANCREATIC JUICE - STERILE ENVIRONMENT										
**Tensile Srength R_m (MPa)**	**arithmetic average**	**Median**	**Min.**	**Max.**	**Lower Quartile**	**Upper Quartile**	**Standard deviation**	**Standard Error**	**Skewness**	**Kurtosis**	**Test Shapiro-Wilka**

**Reference**	236.5	238.1	211.6	273.9	217.4	239.8	21.92	8.95	0.8556	1.2525	p = 0,3933
**7 days**	116.8	118.1	93.1	135.4	109.7	126.3	15.41	6.29	-0.4791	-0.5871	p = 0,6133
**14 days**	55.8	57.0	49.2	60.2	51.4	59.9	4.69	1.91	-0.5229	-1.8839	p = 0,2516
**21 days**	9.3	9.2	7.0	11.1	8.4	10.9	1.58	0.64	-0.2313	-0.7770	p = 0,7725

- The ANOVA test showed a statistically significant difference in the Rm level between all exposure times (**p <0.0001**) (i.e. statistically significant dynamics of Rm changes)
- Tukey's post-hoc test showed a statistically significant difference in the Rm level. There is a statistically significant decrease in the Rm level with the lapse of the exposure time

Table S7. Results of tensile strength tests of Monocryl sutures in the bile (sterile environment).

**MONOCRYL**	**BILE - STERILE ENVIRONMENT**										
**Tensile Srength R_m (MPa)**	**arithmetic average**	**Median**	**Min.**	**Max.**	**Lower Quartile**	**Upper Quartile**	**Standard deviation**	**Standard Error**	**Skewness**	**Kurtosis**	**Test Shapiro-Wilka**
**Reference**	236.5	238.1	211.6	273.9	217.4	239.8	21.92	8.95	0.8556	1.2525	p = 0,3933
**7 days**	123.6	123.2	110.7	136.3	113.5	134.9	10.81	4.41	0.0476	-2.0876	p = 0,4757
**14 days**	70.8	70.1	64.5	77.9	64.6	77.7	6.50	2.65	0.1331	-2.8810	p = 0,0634
**21 days**	16.6	16.2	13.1	20.9	14.9	19.1	2.64	1.00	0.4992	-0.2815	p = 0,8814

Table S8. Results of tensile strength tests of Vicryl sutures in saline (sterile environment).

**VICRYL**	**SALINE - STERILE ENVIRONMENT**										
**Tensile Srength R_m (MPa)**	**arithmetic average**	**Median**	**Min.**	**Max.**	**Lower Quartile**	**Upper Quartile**	**Standard deviation**	**Standard Error**	**Skewness**	**Kurtosis**	**Test Shapiro-Wilka**
**Reference**	293.0	305.8	238.9	320.2	281.0	306.3	29.37	11.99	-1.5789	2.4181	p = 0,0864
**7 days**	252.6	241.5	208.3	309.1	210.7	304.2	44.63	18.22	0.5106	-1.9478	p = 0,1885
**14 days**	208.0	205.3	171.3	241.3	190.0	234.6	28.03	11.44	-0.0329	-1.9912	p = 0,5030
**21 days**	190.9	189.8	155.3	227.3	157.9	225.2	31.32	12.79	0.0764	-1.9307	p = 0,3138
**28 days**	63.0	61.5	51.2	77.5	54.0	72.0	10.96	4.47	0.2573	-2.2627	p = 0,3284

- The ANOVA test showed a statistically significant difference in the Rm level between all exposure times (**p <0.0001**) (i.e. statistically significant dynamics of Rm changes)
- Tukey's post-hoc test showed a statistically significant difference in the Rm level:
  - reference vs day 14 (decrease)
  - reference vs day 21 (decrease)
  - reference vs day 28 (decrease)
  - between day 7 and 21 (decrease)
  - between day 7 vs 28 (decrease)
  - between day 14 and day 28 (decrease)
  - between day 21 and day 28 (decrease)

Table S9. Results of tensile strength tests of Vicryl sutures in the pancreatic juice (sterile environment).

**VICRYL**	**PANCREATIC JUICE - STERILE ENVIRONMENT**										
**Tensile Srength R_m (MPa)**	**arithmetic average**	**Median**	**Min.**	**Max.**	**Lower Quartile**	**Upper Quartile**	**Standard deviation**	**Standard Error**	**Skewness**	**Kurtosis**	**Test Shapiro-Wilka**
**Reference**	293.0	305.8	238.9	320.2	281.0	306.3	29.37	11.99	-1.5789	2.4181	p = 0,0864
**7 days**	240.4	239.2	194.2	284.0	215.7	270.2	36.18	14.77	-0.0527	-2.2352	p = 0,4051
**14 days**	172.4	177.6	141.7	194.0	153.9	190.0	21.60	8.82	-0.4913	-1.7993	p = 0,4291
**21 days**	93.7	89.1	77.9	123.7	82.6	99.8	16.43	6.71	1.4597	2.2465	p = 0,2248

- The ANOVA test showed a statistically significant difference in the Rm level between all exposure times (**p <0.0001**) (i.e. statistically significant dynamics of Rm changes)
- Tukey's post-hoc test showed a statistically significant difference in the Rm level. There is a statistically significant decrease in the Rm level with the lapse of the exposure time

Table S10. Results of tensile strength tests of Vicryl sutures in the bile (sterile environment).

**VICRYL**	**BILE - STERILE ENVIRONMENT**										
**Tensile Srength R_m (MPa)**	**arithmetic average**	**Median**	**Min.**	**Max.**	**Lower Quartile**	**Upper Quartile**	**Standard deviation**	**Standard Error**	**Skewness**	**Kurtosis**	**Test Shapiro-Wilka**
**Reference**	293.0	305.8	238.9	320.2	281.0	306.3	29.37	11.99	-1.5789	2.4181	p = 0,0864
**7 days**	290.7	310.2	186.9	353.0	233.4	350.7	66.96	27.33	-0.8282	-0.8336	p = 0,3065
**14 days**	206.7	210.3	177.0	240.2	177.7	224.9	26.73	10.91	-0.0477	-2.1963	p = 0,3016
**21 days**	123.3	125.0	105.1	138.5	118.2	128.2	11.12	4.54	-0.5664	1.3050	p = 0,7695
**28 days**	19.9	19.7	16.3	23.7	18.9	21.2	2.49	1.02	0.1838	0.7397	p = 0,9617

- The ANOVA test showed a statistically significant difference in the Rm level between all exposure times (**p <0.0001**) (i.e. statistically significant dynamics of Rm changes)
- Tukey's post-hoc test showed a statistically significant difference in the Rm level:
  - reference vs day 14 (decrease)
  - reference vs day 21 (decrease)
  - reference vs day 28 (decrease)
  - between day 7 and 14 (decrease)
  - between day 7 and 21 (decrease)
  - between day 7 vs 28 (decrease)
  - between day 14 and day 21 (decrease)
  - between day 14 and day 28 (decrease)
  - between day 21 and day 28 (decrease)

Table S11. Results of tensile strength tests of PDS sutures in saline (sterile environment).

PDS	SALINE - STERILE ENVIRONMENT										
Tensile Srength R_m (MPa)	arithmetic average	Median	Min.	Max.	Lower Quartile	Upper Quartile	Standard deviation	Standard Error	Skewness	Kurtosis	Test Shapiro-Wilka
**Reference**	117.2	112.7	109.6	132.5	110.5	125.0	9.38	3.83	1.1627	-0.3114	p = 0,0727
**7 days**	108.1	106.4	97.9	120.1	103.0	114.8	8.07	3.30	0.4719	-0.5460	p = 0,8243
**14 days**	113.2	113.9	103.5	122.9	105.0	120.1	7.86	3.21	-0.1343	-1.7220	p = 0,6043
**21 days**	99.2	96.0	87.5	115.9	95.6	104.3	9.75	3.98	0.9912	1.2698	p = 0,3986
**28 days**	98.3	96.1	91.0	112.1	94.3	99.9	7.36	3.00	1.6439	3.1938	p = 0,1434

- The ANOVA test showed a statistically significant difference in the Rm level between all exposure times (**p = 0.0019**) (i.e. statistically significant dynamics of Rm changes)
- Tukey's post-hoc test showed a statistically significant difference in the Rm level:
  - between day 14 vs 21 days (decrease)
  - between day 14 and 28 (decrease)
  - reference vs day 21 (decrease)
  - reference vs day 28 (decrease)

Table S12. Results of tensile strength tests of PDS sutures in the pancreatic juice (sterile environment).

PDS	PANCREATIC JUICE - STERILE ENVIRONMENT										
Tensile Srength R_m (MPa)	arithmetic average	Median	Min.	Max.	Lower Quartile	Upper Quartile	Standard deviation	Standard Error	Skewness	Kurtosis	Test Shapiro-Wilka
**Reference**	117.2	112.7	109.6	132.5	110.5	125.0	9.38	3.83	1.1627	-0.3114	p = 0,0727
**7 days**	100.7	100.9	82.7	113.5	97.0	109.4	10.92	4.46	-0.7265	0.5522	p = 0,7639
**14 days**	88.4	87.8	72.8	111.3	76.0	94.5	13.88	5.67	0.7441	0.5895	p = 0,6358
**21 days**	87.4	88.3	77.0	102.7	77.1	91.2	9.66	3.94	0.4660	0.0535	p = 0,4539
**28 days**	49.3	51.5	38.0	53.4	48.9	52.4	5.75	2.35	-2.06436	1.4503	p = 0,0632

- The ANOVA test showed a statistically significant difference in the Rm level between all exposure times (**p <0.0001**) (i.e. statistically significant dynamics of Rm changes)
- Tukey's post-hoc test showed a statistically significant difference in the Rm level:
  - reference vs day 14 (decrease)
  - reference vs day 21 (decrease)
  - reference vs day 28 (decrease)
  - between day 7 and 28 (decrease)
  - between day 14 and day 28 (decrease)
  - between day 21 and day 28 (decrease)

Table S13. Results of tensile strength tests of PDS sutures in the bile (sterile environment).

PDS	BILE - STERILE ENVIRONMENT										
Tensile Srength R_m (MPa)	arithmetic average	Median	Min.	Max.	Lower Quartile	Upper Quartile	Standard deviation	Standard Error	Skewness	Kurtosis	Test Shapiro-Wilka
**Reference**	117.2	112.7	109.6	132.5	110.5	125.0	9.38	3.83	1.1627	-0.3114	p = 0,0727
**7 days**	117.7	113.3	108.1	139.2	109.0	123.5	11.92	4.87	1.4576	1.6984	p = 0,1309
**14 days**	116.4	115.6	112.3	123.5	114.9	116.4	3.77	1.54	1.5813	3.5622	p = 0,1225
**21 days**	115.7	116.7	106.9	121.1	111.6	121.0	5.56	2.27	-0.7624	-0.4164	p = 0,4624
**28 days**	103.6	104.0	96.8	109.4	98.3	108.7	5.20	2.12	-0.2311	-1.7292	p = 0,4623

- The ANOVA test showed a statistically significant difference in the Rm level between all exposure times (**p <0.0001**) (i.e. statistically significant dynamics of Rm changes)
- Tukey's post-hoc test showed a statistically significant difference in the Rm level:
  - reference vs day 28 (decrease)
  - between day 7 and 28 (decrease)

3. EVALUATION OF THE INFLUENCE OF THE EXPOSURE TIME ON THE TENSILE STRENGTH (Rm) - CONTAMINATED ENVIRONMENT

Table S14. Results of tensile strength tests of Monocryl sutures in the pancreatic juice (infected environment).

MONOCRYL	PANCREATIC JUICE - CONTAMINATED ENVIRONMENT										
Tensile Srength R_m (MPa)	arithmetic average	Median	Min.	Max.	Lower Quartile	Upper Quartile	Standard deviation	Standard Error	Skewness	Kurtosis	Test Shapiro-Wilka
**Reference**	236.5	238.1	211.6	273.9	217.4	239.8	21.92	8.95	0.8556	1.2525	p = 0,3933
**7 days**	116.3	119.0	91.6	132.7	112.1	123.5	14.04	5.73	-1.0940	1.7923	p = 0,5525
**14 days**	26.4	26.3	21.2	32.6	22.3	29.5	4.33	1.77	0.2563	-1.0115	p = 0,8788
**21 days**	5.0	5.1	3.8	6.1	4.6	5.5	0.79	0.32	-0.4306	0.5014	p = 0,9516
**28 days**	236.5	238.1	211.6	273.9	217.4	239.8	21.92	8.95	0.8556	1.2525	p = 0,3933

- The ANOVA test showed a statistically significant difference in the Rm level between all exposure times (**p <0.0001**) (i.e. statistically significant dynamics of Rm changes)
- There is a statistically significant decrease in the Rm level with the lapse of the exposure time.

Table S15. Results of tensile strength tests of Monocryl sutures in the bile (infected environment).

MONOCRYL	BILE - CONTAMINATED ENVIRONMENT										
**Tensile Srength R_m (MPa)**	**arithmetic average**	**Median**	**Min.**	**Max.**	**Lower Quartile**	**Upper Quartile**	**Standard deviation**	**Standard Error**	**Skewness**	**Kurtosis**	**Test Shapiro-Wilka**
**Reference**	236.5	238.1	211.6	273.9	217.4	239.8	21.92	8.95	0.8556	1.2525	p = 0,3933
**7 days**	109.7	114.1	88.5	120.1	105.0	116.3	11.55	4.71	-1.5425	2.2581	p = 0,1525
**14 days**	66.1	67.4	54.6	76.2	60.5	70.7	7.69	3.14	-0.3814	-0.3109	p = 0,9633
**21 days**	13.1	13.5	10.8	15.2	12.0	13.8	1.52	0.62	-0.3588	-0.0376	p = 0,8607
**28 days**	236.5	238.1	211.6	273.9	217.4	239.8	21.92	8.95	0.8556	1.2525	p = 0,3933

- The ANOVA test showed a statistically significant difference in the Rm level between all exposure times (**p <0.0001**) (i.e. statistically significant dynamics of Rm changes)
- There is a statistically significant decrease in the Rm level with the lapse of the exposure time.

Table S16. Results of tensile strength tests of Vicryl sutures in the pancreatic juice (infected environment).

VICRYL	PANCREATIC JUICE - CONTAMINATED ENVIRONMENT										
**Tensile Srength R_m (MPa)**	**arithmetic average**	**Median**	**Min.**	**Max.**	**Lower Quartile**	**Upper Quartile**	**Standard deviation**	**Standard Error**	**Skewness**	**Kurtosis**	**Test Shapiro-Wilka**
**Reference**	293.0	305.8	238.9	320.2	281.0	306.3	29.37	11.99	-1.5789	2.4181	p = 0,0864
**7 days**	161.8	158.3	148.6	184.2	150.6	170.6	13.41	5.47	1.0141	0.2962	p = 0,3711
**14 days**	53.0	56.5	42.8	59.1	44.2	58.9	7.60	3.10	-0.7822	-1.9102	p = 0,0511
**21 days**	15.4	14.8	12.6	18.8	13.3	18.1	2.54	1.04	0.4784	-1.6721	p = 0,4541
**28 days**	293.0	305.8	238.9	320.2	281.0	306.3	29.37	11.99	-1.5789	2.4181	p = 0,0864

- The ANOVA test showed a statistically significant difference in the Rm level between all exposure times (**p <0.0001**) (i.e. statistically significant dynamics of Rm changes)
- There is a statistically significant decrease in the Rm level with the lapse of the exposure time.

Table S17. Results of tensile strength tests of Vicryl sutures in the bile (infected environment).

VICRYL	BILE - CONTAMINATED ENVIRONMENT										
**Tensile Srength R_m (MPa)**	**arithmetic average**	**Median**	**Min.**	**Max.**	**Lower Quartile**	**Upper Quartile**	**Standard deviation**	**Standard Error**	**Skewness**	**Kurtosis**	**Test Shapiro-Wilka**
**Reference**	293.0	305.8	238.9	320.2	281.0	306.3	29.37	11.99	-1.5789	2.4181	p = 0,0864
**7 days**	217.9	224.9	175.6	234.9	214.1	233.1	22.44	9.16	-1.7119	3.0561	p = 0,0506
**14 days**	159.0	160.9	110.1	200.9	128.5	192.9	35.31	14.42	-0.2272	-1.3036	p = 0,6397
**21 days**	42.8	43.9	35.5	45.9	42.3	45.5	3.81	1.55	-1.8598	3.7601	p = 0,0528
**28 days**	293.0	305.8	238.9	320.2	281.0	306.3	29.37	11.99	-1.5789	2.4181	p = 0,0864

- The ANOVA test showed a statistically significant difference in the Rm level between all exposure times (**p <0.0001**) (i.e. statistically significant dynamics of Rm changes)

- There is a statistically significant decrease in the Rm level with the lapse of the exposure time.

Table S18. Results of tensile strength tests of PDS sutures in the pancreatic juice (infected environment).

PDS	PANCREATIC JUICE - CONTAMINATED ENVIRONMENT										
Tensile Srength R_m (MPa)	arithmetic average	Median	Min.	Max.	Lower Quartile	Upper Quartile	Standard deviation	Standard Error	Skewness	Kurtosis	Test Shapiro-Wilka
Reference	117.2	112.7	109.6	132.5	110.5	125.0	9.38	3.83	1.1627	-0.3114	p = 0,0727
7 days	115.3	114.5	107.5	124.4	112.7	118.3	5.67	2.32	0.4601	0.9354	p = 0,9269
14 days	116.0	113.4	102.6	136.2	111.6	118.7	11.21	4.58	1.2253	2.6110	p = 0,3024
21 days	85.6	87.6	63.2	102.6	76.0	96.9	14.69	6.00	-0.5299	-0.7595	p = 0,8142
28 days	63.6	66.5	47.6	72.2	57.3	71.8	9.94	4.06	-0.8656	-0.4635	p = 0,2137

- The ANOVA test showed a statistically significant difference in the Rm level between all exposure times (**p <0.0001**) (i.e. statistically significant dynamics of Rm changes)
- Tukey's post-hoc test showed a statistically significant difference in the Rm level:

- reference vs day 21 (decrease)
- reference vs day 28 (decrease)
- between day 7 and 21 (decrease)
- between day 7 and 28 (decrease)
- between day 14 and day 21 (decrease)
- between day 14 and day 28 (decrease)
- between day 21 and day 28 (decrease)

Table S19. Results of tensile strength tests of PDS sutures in the bile (infected environment).

PDS	BILE - CONTAMINATED ENVIRONMENT										
Tensile Srength R_m (MPa)	arithmetic average	Median	Min.	Max.	Lower Quartile	Upper Quartile	Standard deviation	Standard Error	Skewness	Kurtosis	Test Shapiro-Wilka
Reference	117.2	112.7	109.6	132.5	110.5	125.0	9.38	3.83	1.1627	-0.3114	p = 0,0727
7 days	119.2	119.5	104.2	131.2	112.3	128.7	10.72	4.38	-0.2672	-1.7861	p = 0,5072
14 days	120.2	121.2	114.3	123.6	117.9	123.0	3.65	1.49	-0.8639	-0.3862	p = 0,3349
21 days	106.7	106.4	97.7	113.9	106.0	109.9	5.36	2.19	-0.6538	1.7161	p = 0,5139
28 days	102.3	106.1	87.9	111.1	95.4	107.3	8.81	3.60	-1.0745	-0.0760	p = 0,2089

- The ANOVA test showed a statistically significant difference in the Rm level between all exposure times (**p=0,0014**) (i.e. statistically significant dynamics of Rm changes)
- Tukey's post-hoc test showed a statistically significant difference in the Rm level:

- reference vs day 21 (decrease)
- reference vs day 28 (decrease)
- between day 7 and 21 (decrease)
- between day 7 and 28 (decrease)
- between day 14 and day 21 (decrease)
- between day 14 and day 28 (decrease)
